# Epigenetic Aging Signatures Are Coherently Modified in Cancer

**DOI:** 10.1371/journal.pgen.1005334

**Published:** 2015-06-25

**Authors:** Qiong Lin, Wolfgang Wagner

**Affiliations:** Helmholtz-Institute for Biomedical Engineering, Stem Cell Biology and Cellular Engineering, RWTH Aachen University Medical School, Aachen, Germany; Albert Einstein College of Medicine, UNITED STATES

## Abstract

Aging is associated with highly reproducible DNA methylation (DNAm) changes, which may contribute to higher prevalence of malignant diseases in the elderly. In this study, we analyzed epigenetic aging signatures in 5,621 DNAm profiles of 25 cancer types from The Cancer Genome Atlas (TCGA). Overall, age-associated DNAm patterns hardly reflect chronological age of cancer patients, but they are coherently modified in a non-stochastic manner, particularly at CpGs that become hypermethylated upon aging in non-malignant tissues. This coordinated regulation in epigenetic aging signatures can therefore be used for aberrant epigenetic age-predictions, which facilitate disease stratification. For example, in acute myeloid leukemia (AML) higher epigenetic age-predictions are associated with increased incidence of mutations in *RUNX1*, *WT1*, and *IDH2*, whereas mutations in *TET2*, *TP53*, and *PML-PARA* translocation are more frequent in younger age-predictions. Furthermore, epigenetic aging signatures correlate with overall survival in several types of cancer (such as lower grade glioma, glioblastoma multiforme, esophageal carcinoma, chromophobe renal cell carcinoma, cutaneous melanoma, lung squamous cell carcinoma, and neuroendocrine neoplasms). In conclusion, age-associated DNAm patterns in cancer are not related to chronological age of the patient, but they are coordinately regulated, particularly at CpGs that become hypermethylated in normal aging. Furthermore, the apparent epigenetic age-predictions correlate with clinical parameters and overall survival in several types of cancer, indicating that regulation of DNAm patterns in age-associated CpGs is relevant for cancer development.

## Author Summary

Our genome harbors epigenetic marks, such as DNA methylation (DNAm) at cytosine residues, which govern cellular differentiation. Some epigenetic modifications accumulate throughout life in a highly reproducible manner–they may contribute to the aging process and facilitate reliable age-predictions. So far, little is known how these “epigenetic aging signatures” are modified in cancer tissue and whether or not they are accelerated as compared to normal tissue. In this study, we systematically analyzed age-associated DNAm patterns in many types of cancer. In contrast to non-malignant tissue the epigenetic aging signatures hardly reflect chronological age of cancer patients. This may at least partially be attributed to the fact that cancer is a clonal disease capturing only the epigenetic make-up of the tumor-initiating cell. Notably, the aberrant DNAm patterns are not randomly distributed but reveal co-regulation at regions that become methylated upon aging in non-malignant tissue. Furthermore, we demonstrate that deviations of epigenetic age-predictions correlate with clinical parameters. In fact, they are clearly associated with overall survival in many types of cancer. These findings are particularly important, as they indicate relevance of age-associated DNA methylation patterns for malignant transformation, cancer development and prognosis.

## Introduction

Age is the strongest demographic risk factor for cancer, indicating that molecular changes upon aging trigger malignant transformation. Somatic mutations are usually considered as tumor-initiating events [1]. However, aging is also accompanied by specific epigenetic modifications, which may also contribute to aberrant chromatin conformation and stability [2,3]. Such epigenetic modifications are particularly observed in DNA methylation (DNAm) changes that resemble addition or removal of methyl groups to cytosines in a CpG dinucleotide context. In fact, several CpGs acquire almost linear hypermethylation or hypomethylation upon aging and hence linear univariate or multivariate models can be used to estimate chronological age [4–8]. Such epigenetic age-predictors provide strong biomarkers for biological aging and may support identification of relevant factors for the process of aging—including gender, genetic variants, and body mass index [7,9]. Interestingly, almost the entire set of age-related DNAm changes can be reversed by reprogramming into induced pluripotent stem cells (iPSCs) suggesting that it is possible to reset the aging clock [10,11]. The recent explosion in our knowledge of how chromatin organization modulates gene transcription has further highlighted the importance of epigenetic mechanisms in aging and disease [12].

The cancer epigenome is characterized by simultaneous gains and losses of DNAm throughout the entire genome [13,14]. There is evidence that aberrant DNAm changes at specific sites in the genome–so called epimutations–can mimic somatic mutations to contribute to malignant transformation [15]. Furthermore, it has been suggested that particularly age-associated hypermethylation reveals highly significant overlap with DNAm changes in cancer [16]. Hannum and coworkers used their age-predictor on DNAm data from The Cancer Genome Atlas (TCGA) data portal of matched samples of cancer tissue and normal tissue and found epigenetic age seemingly accelerated in cancer [7]. On the other hand, a systematic analysis using another age-predictor by Horvath, also known as epigenetic clock, indicated that several tumors exhibit negative age acceleration [6]. Thus, age-related DNAm patterns are affected in malignant diseases, but it is yet unclear how they are regulated and whether or not they reflect cell division numbers of long-lived cell populations in cancer.

In this study we followed the hypothesis that the age-related DNAm patterns are coordinately regulated in cancer. Furthermore, we analyzed if epigenetic aging signatures are associated with clinical parameters and prognosis. To address these questions we used available DNAm datasets of 25 different tumor entities from the TCGA data portal. All of these profiles have been analyzed using the Illumina 450k BeadChip, which represents more than 480,000 CpGs [17].

## Results

### Age-associated hypermethylation is coherently modified in AML

Initially, we focused on acute myeloid leukemia (AML) that has been demonstrated to reveal pronounced changes in the epigenetic landscape [18–21]. Advantages of choosing this tumor were that AML comprises relatively high percentages of malignant cells that can be estimated by blast counts and the availability of large DNAm datasets of whole blood derived from healthy controls that be used as a reference. To identify age-related CpGs in normal blood we used the dataset of Hannum and coworkers, consisting of DNAm profiles of 656 human individuals (19 to 101 years old) [7]. 432 CpGs revealed clear correlation of DNAm levels and aging: 94 CpGs were hypermethylated and 338 CpGs were hypomethylated (Spearman correlation ρ > 0.5 or ρ < -0.5, respectively). In line with previous studies, age-related hypermethylation was significantly enriched in CpG islands (CGIs) and gene promoter regions, whereas hypomethylation occurs rather outside of CGIs and of gene promoters (S1 Fig) [22–24].

**Fig 1:**
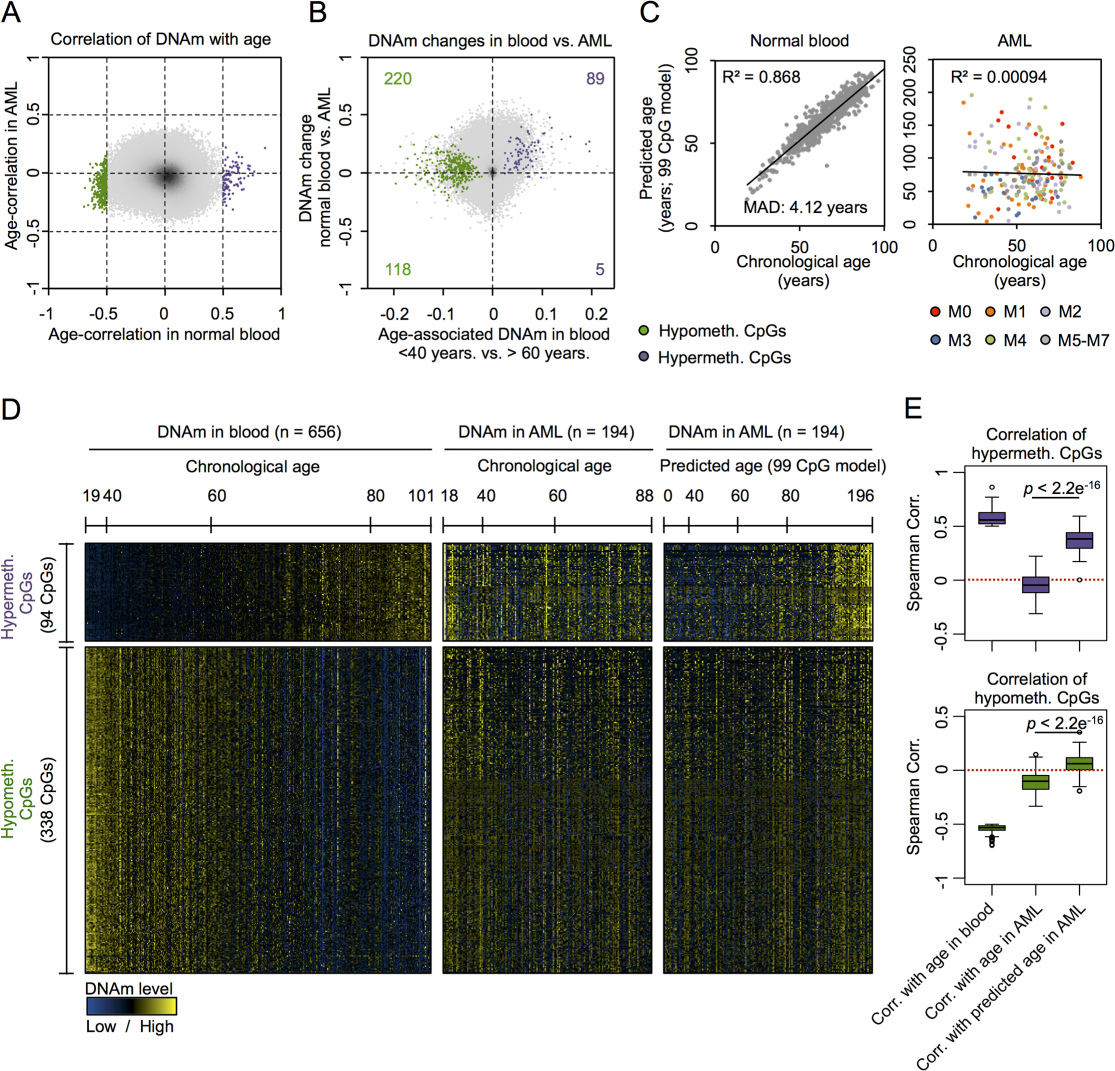
CpGs with age-associated hypermethylation are coherently modified in AML. (A) DNAm levels at age-associated CpGs revealed clear correlation in normal blood (94 hypermethylated and 338 hypomethylated; Spearman’s rank correlation ρ > 0.5 or ρ < -0.5) [**7**], whereas this was not observed for any CpG site in AML samples [**25**]. (B) All CpGs with age-associated hypermethylation were in average higher methylated in AML as compared to normal blood (chi-square *p*-value = 1.8 * 10^**−8**^). (C) A multivariate model based on 99 age-associated CpGs (99 CpG model) [**8**] can reliably predict chronological age in blood, but not in AML (color code represents FAB classification). (D) Heatmap analysis of age-associated CpGs in blood and AML: in AML patterns do not correlate with chronological age, but with epigenetic age-predictions (99 CpG model). (E) CpGs with age-associated hypermethylation do not correlate with chronological age in AML, but there is a highly significant association with predicted age. In contrast, there was no correlation with predicted age in hypomethylated CpGs (Wilcoxon rank-sum test).

Next, we performed the same analysis on 194 DNAm profiles of AML samples from TCGA (18 to 88 years old) [25]. We did not observe a single CpG site that revealed clear correlation with chronological age of the patients using the same arbitrary and relatively stringent cutoff (Fig 1A). This was unexpected, as we initially supposed that at least some of the age-related CpGs reside in chromosomal areas that are not affected by malignant transformation–and hence reflect the chronological age of the patients. Analysis of the subset of AML samples with less than 60% blasts demonstrated correlation of DNAm at several CpGs with chronological age—and this was not observed in the subset with more than 82% blasts (S2 Fig). However, there was no overlap of age-associated CpGs in AML with low blast counts and normal blood. This may at least partly be attributed to differences between peripheral blood (normal control) *versus* bone marrow aspirates (AML samples) or differences in cellular composition. Overall, DNAm levels were increased in AML as compared to normal blood, and this was particularly observed in CpG sites with age-associated hypermethylation (chi-square *p*-value = 1.8 * 10^−8^) but not for CpGs with age-associated hypomethylation (Figs 1B and S3A).

To further analyze age-associated DNAm changes in AML we used our previously described epigenetic age-predictor, which has been developed on various other DNAm datasets of blood samples and comprises 99 CpGs, subsequently referred to as “99 CpG model” [8]. A multivariate model with these 99 CpGs facilitated reliable age-predictions in the Hannum dataset of normal blood with a mean absolute deviation (MAD) of only 4.12 years (Pearson correlation *R* ^2^ = 0.87) [8]. In contrast, the same model did not reveal any correlation between chronological and predicted age of AML profiles (Fig 1C; *R* ^2^ < 0.001) [3]. In average the predicted age of AML patients was 21 years older than their chronological age, but there were also several AML patients which were predicted to be younger. Heatmap analysis of those CpGs that reveal age-associated changes in normal blood provided further evidence that DNAm patterns in AML are not associated with the chronological age of the patients (Fig 1D).

This led us to the hypothesis that age-associated DNAm patterns might be modified coherently within AML samples—independent from the aging process. In fact, there was a clear relationship between DNAm patterns at age-associated CpGs, as identified above in normal blood, with epigenetic age-predictions using the 99 CpG model—even though these sets of CpGs were independent. Notably, this relationship of DNAm patterns in AML with epigenetic age-predictions was almost exclusively observed in CpGs that revealed age-associated hypermethylation in normal tissue, but not in CpGs with age-associated hypomethylation (Fig 1D and 1E). To further substantiate these findings we alternatively used the age-predictor by Horvath, which is based on 375 CpGs and applicable for different tissues [6]. The two different age-predictors revealed similar results in AML (S3B Fig; *R* ^2^ = 0.71)—even though predictions of both methods did not correlate with chronological age of the AML patients—and this supports the notion that age-related DNAm patterns are not randomly affected [3]. Furthermore, using the Horvath-predictor we observed the same trend: DNAm patterns at CpGs that become normally hypermethylated upon aging clearly correlated with epigenetic age-predictions in AML, whereas this was not observed for hypomethylated CpGs (S3C and S3D Fig). These results indicate that DNAm changes are coordinately modified within AML samples, particularly at CpGs that usually reveal age-associated hypermethylation.

### DNAm patterns in age-associated CpGs correlate with clinical parameters

**Fig 2:**
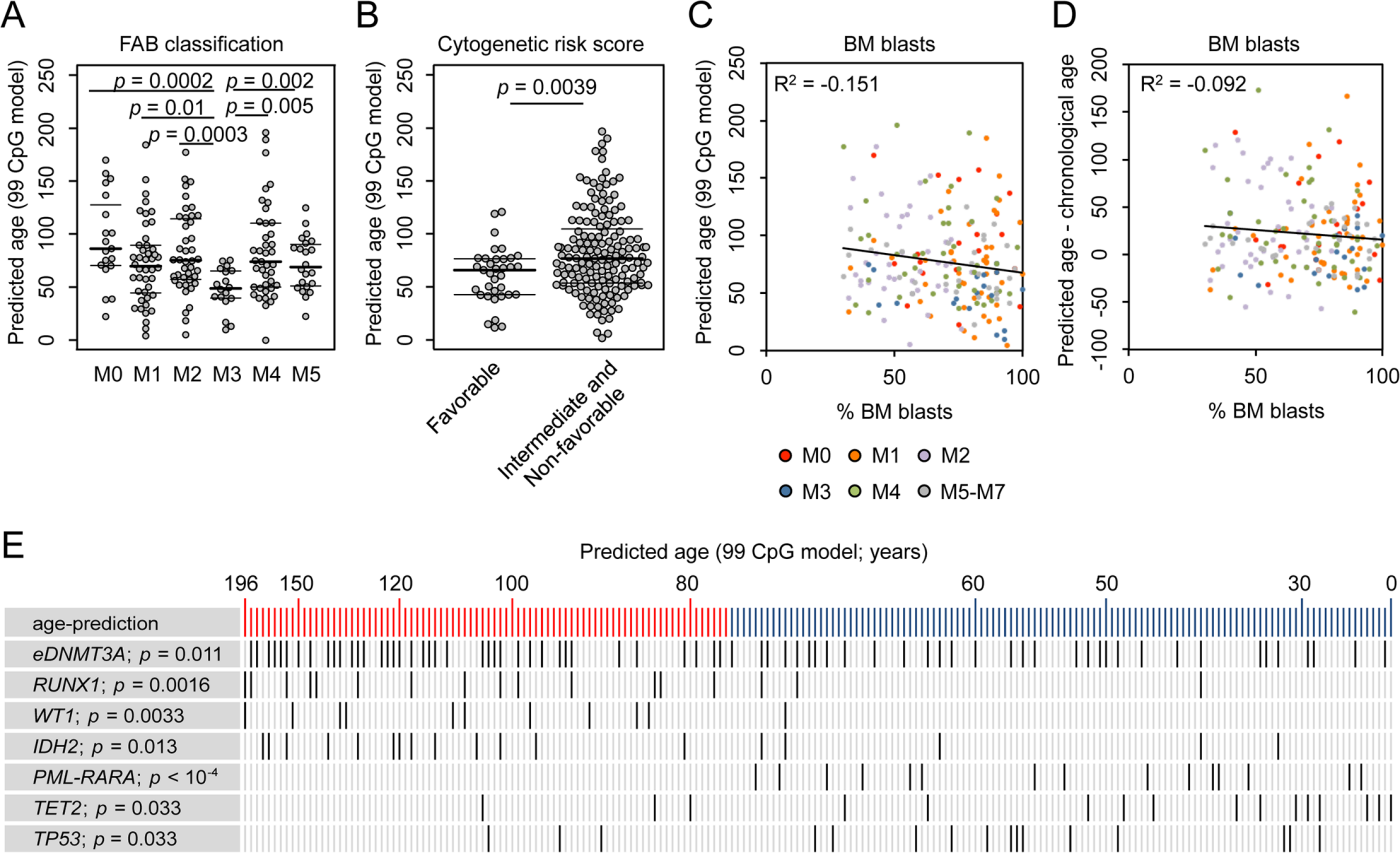
Correlation of epigenetic aging signatures with clinical parameters in AML. (A) Epigenetic age-predictions were younger in acute promyelocytic leukemia (APL; FAB classification M3) in comparison to other AML subtypes. (B) Patients with favorable cytogenetic risk score revealed a younger epigenetic phenotype than other AML samples (intermediate or non-favorable risk score; Wilcoxon rank-sum test, *p* = 0.0039). (C) The percentage of bone marrow (BM) blasts did not correlate with epigenetic age-predictions (99 CpG model; color code represents FAB classification). (D) Furthermore, the deviation of chronological and predicted age was not related to blast counts, too. Even samples with less than 50% BM blasts reveal clear offset in epigenetic age-predictions. (E) AML patients were ranked according to epigenetic age-predictions and mutations in the corresponding genes are indicated (*eDNMT3A* = epimutation in *DNMT3A* [**15**]; *PML-PARA* = translocation that characterizes APL; Student´s t-test).

If age-related DNAm patterns are consistently changed in AML–either indicative for positive or negative epigenetic age-acceleration–then these changes might also be associated with clinical parameters as provided by TGCA consortium (S1 Table) [25]. We found that patients with acute promyelocytic leukemia (APL; FAB classification M3) had significantly younger epigenetic age-predictions, which might also reflect the fact that APL rather occurs in younger patients than the other subtypes of AML (Fig 2A) [26]. Furthermore, AML patients with favorable cytogenetic risk score were rather predicted to be younger than the rest (Fig 2B; Wilcoxon rank-sum test: *p* = 0.0039). The number of bone marrow blasts correlated neither with epigenetic age-predictions (Fig 2C) nor with the deviation of predicted and chronological age (Fig 2D). This might be due to the fact that even a subset of 30% malignant cells can greatly interfere with the models for epigenetic age-predictions because age-associated DNAm changes at individual CpGs are often rather small. Furthermore, the number of mutated genes (S4A Fig), or gender (S4B Fig) did not reveal obvious correlation with epigenetic age-predictions. To determine, whether or not epigenetic age-predictions in AML might be affected by blood cell type composition we estimated the relative proportions using the algorithm developed by Houseman et al. [27,28]: epigenetic age-predictions in AML samples did not correlate with predictions of any cell type (S4C Fig). These findings support the notion that age-associated changes are independent of changes in blood cell type composition [29]—although this approach was developed for normal blood and may be impaired by aberrant DNAm profiles in AML.

We hypothesized that changes in the pattern of age-associated DNAm might also be relevant for prognosis but this was not evident for AML: Kaplan-Meier plots did not reveal significant differences in overall survival (OS) of patients predicted to be either younger or older than mean age-predictions (S5 Fig; 99 CpG model: *p* = 0.198; Horvath-predictor: *p* = 0.127). Furthermore, it can be expected that chronological age is a cofounder for analysis of OS, as it is well known that prognosis is better in younger AML patients (S5D Fig; *p* = 0). When we used multivariate Cox regression analysis to adjust for chronological age the relevance for predicted age did not become clear, too (99 CpG model: *p* = 0.47; Horvath-predictor: *p* = 0.82), indicating that age-related epigenetic signatures may not provide an indicator for OS in AML.

To further analyze whether or not epigenetic age-predictions are reflected on gene expression level we used corresponding RNA-seq data of TCGA: 11 genes were differentially expressed between patients predicted to be younger or older than mean age-predictions (S2 Table; *C7orf13*, *CLU*, *DSC2*, *FAM127A*, *FAM127B*, *JAG1*, *LOC644538*, *NHLRC1*, *TEKT2*, *and TTC12* were higher expressed in older group*; NEXN* was less expressed; adjusted *p*-value < 0.05) and this did not seem to be evoked by corresponding changes in DNAm patterns.

AML is a very heterogeneous disease. Specific mutations are associated with marked differences DNAm patterns and this might interfere with our analysis [18–21]. Therefore, we separately analyzed AML subsets with specific mutations: even within these subsets DNAm patterns were not coupled with chronological age, whereas DNAm levels at CpGs that are normally hypermethylated upon aging correlate well with epigenetic age-predictions (S6 Fig). Subsequently, we analyzed if specific somatic mutations are overall associated with higher or lower epigenetic age-predictions (Fig 2E and S3 Table). In fact, mutations within the genes runt-related transcription factor 1 (*RUNX1; p* = 0.0016), Wilms tumor 1 (*WT1; p* = 0.0033), and isocitrate dehydrogenase 2 (*IDH2*; *p* = 0.013) revealed significantly higher incidence in AMLs with enhanced epigenetic aging. Epimutations in *DNMT3A* (*eDNMT3A*), defined by aberrant high DNAm level within an internal promoter region of *DNMT3A* [15], were also more frequent in samples with older age-predictions (*p* = 0.011) as described before [3]. In contrast, mutations in ten eleven translocation 2 (*TET2*; *p* = 0.033) and tumor protein p53 (*TP53; p* = 0.033), as well as the *PML-PARA* translocation (*p* = 6.7 * 10^−5^), which fuses the retinoic acid receptor alpha (RARA) gene on chromosome 17 with the PML gene on chromosome 15 (characteristic for APL), are significantly enriched in AMLs with younger age-predictions. These results indicate that specific mutations are somehow related to age-associated DNAm patterns [3,6]–but it is yet unclear if they are cause or consequence.

### Age-associated DNAm changes across different cancer types

**Fig 3:**
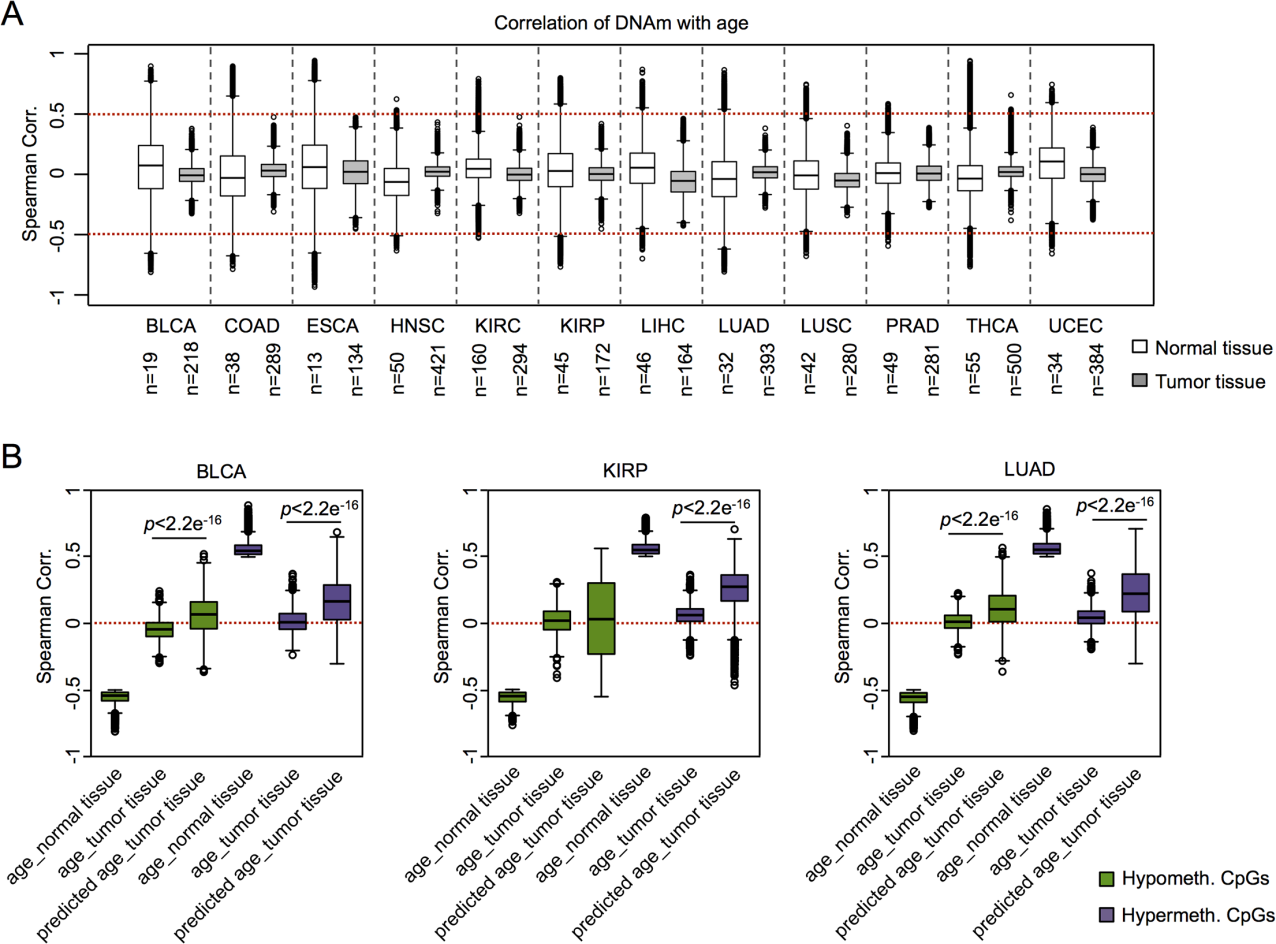
Age-associated DNAm is not related to chronological age in cancer. (A) Age-associated CpGs (ρ > 0.5 or ρ < -0.5) were identified in datasets of TCGA for normal control tissues, whereas this was not observed in corresponding tumors. (B) CpGs that reflect age-associated hypomethylation (green) or hypermethylation (purple) in the corresponding normal tissues were further analyzed in cancer tissue: hypermethylated regions correlated rather with epigenetic age-predictions (Horvath-predictor) than with chronological age. Results for bladder urothelial carcinoma (BLCA), kidney renal papillary cell carcinoma (KIRP), and lung adenocarcinoma (LUAD) are exemplarily depicted (Wilcoxon rank-sum test).

Subsequently, we addressed the question whether or not coherent modification of epigenetic aging signatures was also observed in other types of cancer. For this analysis we had to take into account that there are tissue-specific differences in age-associated DNAm [30–32]. For cross-tissue comparison we therefore analyzed twelve DNAm datasets from the TCGA data portal, which included reference samples from normal tissue. Initially, we focused on the above mentioned age-associated CpGs in normal blood (as depicted in Fig 1A) [7]: in normal tissues CpGs with age-associated hypermethylation in blood revealed overall also age-associated hypermethylation in other tissues, whereas this did not apply to hypomethylated CpGs. Furthermore, age-associated DNAm changes in normal blood were not recapitulated by any malignant tissue (S7 Fig). Subsequently, we selected age-associated DNAm changes for each individual dataset of control tissue and identified many CpGs that clearly correlated with the chronological age of the donor, including hyper- and hypomethylated CpGs (ρ > 0.5 or ρ < -0.5, respectively). In contrast, almost none of these CpGs revealed correlation with chronological age in the corresponding malignant tissue (Fig 3A). These results demonstrate that age-associated DNAm changes are overall decoupled from the chronological age in cancer.

To gain further insight into DNAm patterns at CpGs which are usually age-associated we used epigenetic aging signatures (99 CpG model or the Horvath-predictor) on all 25 cancer types of TCGA. As expected, age-predictions hardly correlated with chronological age in any type of cancer (Table 1). Furthermore, cancer tissue did not always reflect enhanced age-associated changes–in many types of cancer age-predictions rather suggested negative age-acceleration, particularly when using the Horvath-predictor, as described before (S8 Fig) [6]. Notably, epigenetic age-predictions correlated when using the two independent epigenetic aging signatures (99 CpG model and Horvath-predictor) in all types of cancer–even though the 99 CpG model was only trained on blood. Only for prostate adenocarcinoma (PRAD; *R* ^2^ = 0.40) and rectum adenocarcinoma (READ; *R* ^2^ = 0.30) the correlation was relatively low (S4 Table).

**Table 1:** Association of overall survival with epigenetic age-predictions.

Tumor type	Short cut	Number of DNAm profiles	Corr. age vs. predicted age	Univariate Cox regression p-value	Multivariate Cox regression		
					p-value (all)	p-value (age)	p-value (predicted age)
Thyroid carcinoma	THCA	500	0.64	**0.002**	**7.00E-08**	**5.20E-06**	0.94
Kidney chromophobe	KICH	66	0.37	**0.007**	**0.022**	0.49	**0.019**
Esophageal carcinoma	ESCA	134	0.42	**0.013**	**0.025**	0.28	**0.011**
Lower grade glioma	LGG	412	0.61	**0.024**	**2.00E-14**	**5.20E-15**	**0.001**
Pheochromocyt. & paraganglioma	PCPG	97	0.33	**0.030**	0.062	0.35	**0.024**
Renal clear cell carcinoma	KIRC	294	0.43	**0.050**	**0.006**	0.012	0.45
Pancreatic adenocarcinoma	PAAD	79	0.35	0.058	0.166	0.95	0.088
Lung squamous cell carcinoma	LUSC	280	0.35	0.100	**0.021**	0.032	**0.027**
Skin cutaneous melanoma	SKCM	358	0.23	0.146	**1.80E-06**	**1.20E-06**	**0.007**
Uterine carcinosarcoma	UCS	57	0.06	0.206	0.142	0.12	0.25
Lung adenocarcinoma	LUAD	393	0.23	0.291	0.443	0.47	0.23
Colon adenocarcinoma	COAD	289	0.21	0.365	0.447	0.38	0.56
Sarcoma	SARC	115	0.14	0.368	0.29	0.2	0.27
Glioblastoma multiforme	GBM	127	0.32	0.406	**1.20E-07**	**1.60E-07**	**0.003**
Uterine corpus endometrial CA	UCEC	384	0.15	0.457	0.126	0.06	0.27
Bladder urothelial carcinoma	BLCA	218	0.07	0.460	0.328	0.2	0.48
Liver hepatocellular carcinoma	LIHC	164	0.24	0.461	0.762	0.97	0.47
Prostate adenocarcinoma	PRAD	281	0.29	0.481	0.462	0.33	0.39
Rectum adenocarcinoma	READ	96	0.32	0.552	0.28	0.17	0.81
Acute myeloide leukemia	LAML	194	-0.02	0.560	**1.70E-08**	**1.70E-08**	0.82
Endocervical adenocarcinoma	CESC	195	0.1	0.671	0.154	0.058	0.91
Adrenocortical carcinoma	ACC	78	0.49	0.736	0.826	0.6	0.58
Head and neck squamous cell CA	HNSC	421	0.15	0.844	**0.031**	**0.0092**	0.6
Stomach adenocarcinoma	STAD	295	0.05	0.846	0.683	0.4	0.8
Kidney renal papillary cell CA	KIRP	172	0.31	0.951	0.934	0.72	1

Significant values (p < 0.05) for univariate and multivariate Cox regression analysis (only adjusted for chronological age) are indicated in bold. Predicted age was calculated using Horvath-predictor. CA, carcinoma.

We then analyzed if, in analogy to AML, coherent changes with epigenetic aging signatures are particularly observed in CpGs with age-associated hypermethylation. To this end, we focused on CpGs with age-associated changes in the corresponding normal tissue and analyzed their correlation of DNAm levels in cancer tissue–either with chronological age of the patient or with predictions by the epigenetic aging signatures. In fact, DNAm levels at hypermethylated CpGs were associated with epigenetic age-predictions rather than with chronological age across almost all tumors, whereas age-associated hypomethylation did neither correlate clearly with chronological age nor with predicted age (Fig 3B). Only for colon adenocarcinoma (COAD) the opposite tendency was observed (S5 Table). Thus, DNAm patterns in CpGs, which are hypermethylated upon aging in normal tissue, are coherently modified in almost all cancer types analyzed.

### Epigenetic aging signatures are associated with overall survival in several tumors

**Fig 4:**
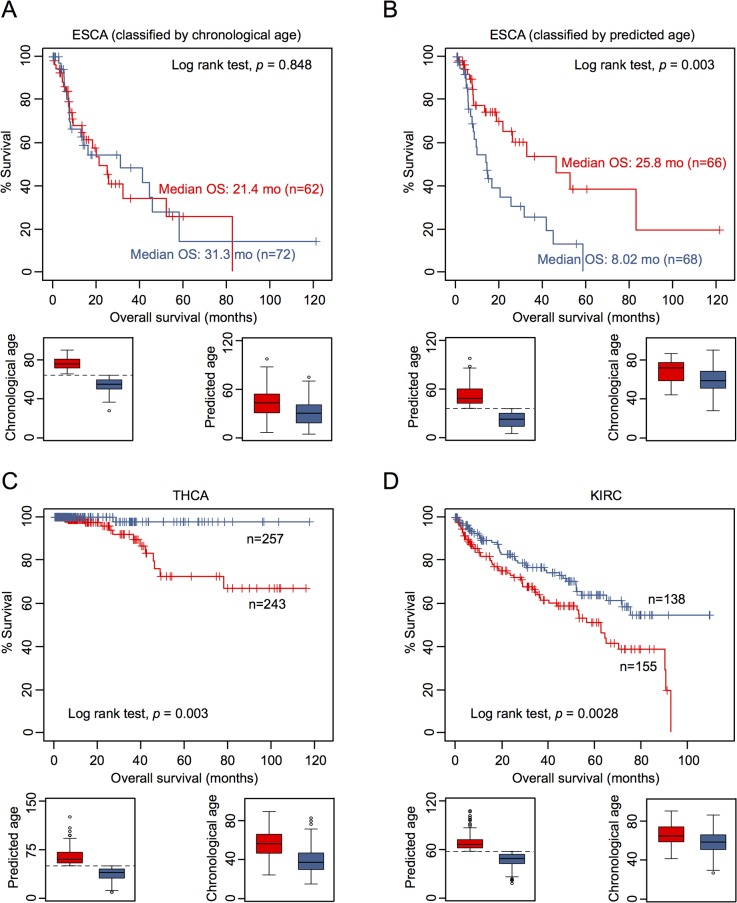
Epigenetic aging signatures are indicative for survival in several tumors. (A) Kaplan-Meier plots demonstrate overall survival between esophageal carcinoma (ESCA) patients classified by chronological age (red: older than mean age of all patients; blue: younger). (B) Alternatively, the datasets were classified by epigenetic age-predictions (Horvath-predictor, red: older than mean age-predictions of all patients; blue: younger). Younger epigenetic age-predictions were associated with poorer prognosis—even thought this was not observed with regard to chronological age. In analogy, DNAm profiles of (C) thyroid carcinoma (THCA) and (D) renal clear cell carcinoma (KIRC) were stratified by mean age-predictions and in these tumors younger epigenetic age-predictions were associated with better prognosis.

To determine whether or not DNAm patterns in epigenetic aging signatures are associated with clinical outcome we have classified DNAm profiles according to the mean age-prediction (Horvath-predictor). Kaplan-Meier estimation revealed that patients with esophageal carcinoma (ESCA) have a significant better overall survival (*p* = 0.003) if predicted to be older, whereas this was not observed with regard to chronological age (Fig 4A and 4B). On the other hand, patients with thyroid carcinoma (THCA; *p* = 0.003; Fig 4C) and with renal clear cell carcinoma (KIRC; *p* = 0.008; Fig 4D) had a better prognosis if predicted to be younger. Furthermore, in tendency glioblastoma multiforme had better OS if predicted to be older (GBM; *p* = 0.059), whereas patients with kidney chromophobe cancer had better prognosis if predicted to be younger (KIRH; *p* = 0.052; S9 Fig).

Subsequently, we performed univariate Cox regression analysis as well as multivariate Cox regression analysis, which was only adjusted for chronological age. In fact, epigenetic aging signatures were clearly associated with overall survival, independent from chronological age, in several tumors (Table 1; KICH, ESCA, LGG, LUSC, PCPG, SKCM, and GBM). Please note that better outcome was inconsistently attributed either to younger or older epigenetic age-predictions in the different tumor types.

It is yet unclear if methylation-based age-predictors provide independent risk factors in any of these tumors. However, we have exemplarily tested association of some other clinical parameters with epigenetic age-predictions in ESCA. In fact, ESCA patients were predicted to be younger if they were male or had the squamous cell carcinoma subtype (S10 Fig). Subsequently, we calculated a multivariate cox model for ESCA, adjusting for chronological age, predicted age, gender, smoking, histologic diagnosis, and tumor grade. Using all of these parameters the model was overall not predictive (*p* = 0.135), whereas epigenetic age-prediction seemed to be the only parameter with predictive power (*p* = 0.021; S6 Table).

## Discussion

In this study we demonstrate that DNAm patterns in cancer, albeit not related to chronological age, are modified in a combined fashion at CpGs, which become hypermethylated upon aging in non-malignant tissue. Furthermore, we provide compelling evidence that these modifications can be implemented for disease stratification and assessment of prognosis in several cancers. Age-associated DNAm changes are normally observed genome wide [4,7] and we initially anticipated that they are preserved at least in some regions of the cancer epigenome. However, none of the CpGs revealed clear correlation with chronological age across all 25 different sets of cancer analyzed–even though tumor tissue comprises also non-malignant cells, such as blood cells or vessel cells, which drive age-predictions towards the chronological age. This might also be the reason why particularly for AML samples, which often comprise high proportions of blasts, the correlation between predicted age and chronological age was extremely low. Importantly, cancer is a clonal disease and therefore malignant cells capture only the epigenetic make-up of an individual cell, whereas β-values in normal tissue may reflect a cross-section of various different stages of cellular aging. In this regard epigenetic aging signatures in cancer do not reflect accelerated or decelerated rates of aging—they would rather reflect the state of aging in the tumor-initiating cell, unless the corresponding DNAm pattern is modified thereafter.

Regulation of age-associated DNAm patterns seems to differ in hypermethylated and hypomethylated CpGs. It has been shown that age-associated hypermethylation is enriched in CpG islands (CGIs) and shore regions, whereas hypomethylation occurs rather outside of CGIs [8,22,33–36] and this has been further substantiated by our analysis. Age-associated hypermethylation occurs preferentially at developmental gene promoters that bear distinctive bivalent chromatin signatures in stem cells, and that are frequently hypermethylated in various cancers [31]. Furthermore, promoters in polycomb group proteins target genes (PCGTs) are more likely to be methylated both in aging [16] and cancer [37,38]. Notably, similar gains and losses of DNAm were also observed in replicative senescence [10,39]. In this study, we demonstrate that age-associated hypermethylation, as identified in blood, is conserved across several normal tissues, whereas this was not evident for age-associated hypomethylation. This is in line with previous studies demonstrating that age-related changes between different cell types are more consistent in hypermethylated CpGs [31]. Furthermore, we demonstrate that particularly CpGs with age-associated hypermethylation seem to be coherently modified in cancer. These results suggest that a molecular mechanism—e.g. mediated by polycomb complexes, non-coding RNAs, or modifications of the histone code [40]—drives DNAm changes at CpGs with age-associated hypermethylation. It is also well conceivable, that the significant enrichment of the hypermethylated CpGs within CGIs is relevant for this regulative process. In contrast, age-associated hypomethylation seems to be rather evoked by stochastic epigenetic drift [4,41]. The fact that age-associated DNAm is reversed during reprogramming into iPSCs indicates that this process can be controlled *per se*—but it is yet unclear if these DNAm changes are really functionally relevant [6,8].

So far, our understanding of the mechanisms causing somatic mutations in specific types of cancer is remarkably limited [42]. Changes in the chromatin conformation, which may also be triggered by DNAm, contribute to mutational hotspots [12,43]. For example, it has been shown that aberrant hypermethylation in the *p16Ink4a* promoter increased incidence of spontaneous cancer in mice [44]. We demonstrated that mutations in *RUNX1*, *WT1*, and *IDH2* are more frequent in patients with increased age-predictions, whereas mutations in *TET2* and *TP53* have higher incidence with younger age-predictions. The latter is in line with findings by Horvath, demonstrating that mutations in *TP53* are more frequent in epigenetically young samples across many types of cancers [6]. Differential methylation in cytogenetically normal AML was specifically found in genes encoding transcription factors, particularly within the gene *WT1* [45]. AML subsets with mutations in *IDH1/2* or *TET2* revealed related hypermethylation signatures [21]. Furthermore, age-associated hypermethylation within *TET2* has been described in epidermis [46]. In this regard, age-associated DNAm changes may contribute to specific mutations or *vice versa*. Rather than separating genetics from epigenetics, or try to decide which is more important for cancer initiation, the past few years have emphasized that these fields are merging [13]. It might be speculated that reversal of age-associated DNAm changes in cancer contributes to immortalization—and hence favors malignant transformation. Thus, age-associated alterations of the DNAm pattern may resemble a double edged sword: they may provide an anti-proliferative barrier for aging cells to prevent cancer initiation, but they may also favor changes in chromosomal organization that trigger other mutations. This might also be a reason why enhanced epigenetic aging was associated with better prognosis in some tumor types and with worse prognosis in others.

Aging is associated with highly reproducible DNAm changes in normal tissue. However, in cancer these DNAm patterns hardly reflect chronological age of the patient. Epigenetic age-predictions are not always overestimated in malignant diseases. In fact, they are often predicted to be younger [7,24]. Therefore, aberrant DNAm at these regions can not only be attributed to accelerated epigenetic aging caused by higher cell proliferation. The epigenetic make-up rather captures the state of epigenetic aging of the tumor-initiating cell or it is coherently modified by an underlying epigenetic process—the latter is more likely because this association is particularly observed in hypermethylated CpGs across different types of cancer. Furthermore, our analysis unequivocally demonstrates that DNAm patterns at age-associated CpGs are related to overall survival or other clinical parameters in a disease specific manner. Aberrant DNAm can predispose to malignancy [47] and it is therefore tempting to speculate that modification of age-associated DNAm patterns in tumor initiating cells acts as one of multiple “hits” required for cancer development in the elderly.

## Methods

### Datasets used in this study

We utilized a large number of DNAm datasets generated on the HumanMethylation450K BeadChip platform [17]: DNAm profiles of 656 normal blood samples were downloaded from NCBI GEO (GSE40279) [7]. We retrieved all at the time available DNAm data from the TCGA data portal (http://cancergenome.nih.gov/), which provides a unique and well-curated collection of molecular profiles of many cancer entities. Corresponding clinical parameters—including chronological age, tumor subtypes, classifications, mutations, survival data etc.—were also retrieved from TCGA. Our compilation comprised 5,621 DNAm profiles for 25 different types of cancer with information on chronological age of the patient (Table 1). Furthermore, 12 of these datasets included control samples (n > 10) from matched non-tumorous adjacent tissue (in total 570 control samples). DNAm profiles reflect methylation-levels at more than 480,000 CpGs on single nucleotide resolution as “β-values” ranging from 0 (non-methylated) to 1 (100% methylation) [17].

### Definition of age-related CpGs

Age-related CpGs were identified by correlation of β-values with chronological age in non-tumorous tissue using Spearman’s rank correlation (ρ). For selection of hyper- or hypo-methylated CpGs we used a cutoff of ρ > 0.5 or ρ < -0.5, respectively. In contrast to other studies [7] we did not adjust for other variables and have therefore chosen this relatively stringent cutoff. This analysis was performed for each of the datasets individually. It has been demonstrated that several probes on the Illumina 450k Bead Chip overlap with known SNPs or might be cross-reactive [48], but these were not excluded for this study because the published age-predictors comprised such CpGs. Heatmaps of DNAm profiles were plotted using R.

### Epigenetic age-predictions

Two independent epigenetic age-predictors were used in this study: The 99 CpG model has been established on various DNAm profiles of normal blood samples (GSE19711, GSE20242, GSE20242, GSE23638, GSE20236, and GSE27317; all HumanMethylation27K BeadChip) and then modified for the 450K BeadChip as described before [8]. Briefly, the 99 age-related CpG sites were implemented in a multivariate linear model that was trained and tested by leave one out cross validation method using other DNAm data sets from normal blood in R caret package (http://cran.r-project.org/web/packages/caret/). Additionally, we applied Horvath-predictor using a penalized linear regression model with 354 CpG sites to predict the age of multiple tissues [6]. Our analysis was performed with or without normalization of β-values–as the results were very similar we only present results with non-normalized data. Both age-predictors were applied to each of the 1,226 DNAm profiles of control tissue and 5,621 DNA profiles of cancer tissue. Subsequently, we analyzed Spearman’s rank correlation of β-values against these epigenetic age-predictions.

### Comparison of epigenetic age-predictions with clinical parameters

Association of clinical parameters (e.g. tumor classification, risk scores, and gender) with either chronological age or epigenetic age-predictions was estimated by Wilcoxon rank-sum test (*p* < 0.05 was considered significant). Overall survival (OS) was analyzed in all cancer samples based on TCGA information. For AML, we additionally used event free survival (EFS), defined by the duration from start of the treatment to either disease progression or death (regardless of cause of death) [49]. Cancer samples were stratified into two groups according to the geometric mean of age-predictions. The survival data in these groups was compared using the log-rank test and visualized by Kaplan-Meier survival plot using R package survival (http://cran.r-project.org/web/packages/survival/). Furthermore, we used the Cox Proportional Hazards Model, which does not require upfront classification, both as univariate (epigenetic age) or multivariate cox regression model (chronological age + epigenetic age; unless mentioned otherwise) to estimate the prognostic importance of epigenetic age. For comparison of epigenetic age-predictions with incidence of somatic mutations we used the analysis of whole-genome sequencing (n = 50) or whole-exome sequencing data (n = 150) of AML samples from TCGA [25]. Samples were classified by mean age-predictions into two groups and Student´s t-test was used to compare the frequency of mutations in individual genes between the two groups (*p* < 0.05).

### Gene expression profiles of AML

Processed RNA-sequencing data of AML patients (n = 173) were retrieved from TCGA. The mRNA level of each gene is estimated by the RPKM values (read per kilobase of exon per million mapped reads). Data were log2 transformed and quantile normalized for further analysis. The differentially expressed genes between two groups, as classified by geometric mean of age-predictions, were identified using limma t-test in R (adjusted *p*-value < 0.05 and fold change > 2).

## Supporting Information

S1 Fig Enrichment of age-associated CpGs in genomic context. (PDF)

S2 Fig Age-associated DNAm changes in AML samples with high or low blast counts. (PDF)

S3 Fig Age-associated DNAm changes in AML. (PDF)

S4 Fig Association of epigenetic age-predictions with clinical parameters in AML. (PDF)

S5 Fig Association of epigenetic age-predictions with clinical outcome in AML. (PDF)

S6 Fig DNAm of age-associated CpGs in AML samples with *IDH2* or *WT1* mutations. (PDF)

S7 Fig Age-associated hypermethylation in blood is also reflected in other tissues. (PDF)

S8 Fig Deviation of epigenetic age-predictions in various types of cancer. (PDF)

S9 Fig Kaplan-Meier analysis of glioblastoma multiforme and kidney cancer. (PDF)

S10 Fig Epigenetic age-predictions in esophageal carcinoma. (PDF)

S1 Table Clinical information about AML patients (TCGA). (PDF)

S2 Table Differentially expressed genes in AML samples predicted young vs. old. (PDF)

S3 Table Epigenetic age-predictions of AML samples with specific somatic mutations. (PDF)

S4 Table Correlation of age-predictions using 99 CpG model and Horvath-predictor for different cancer types. (PDF)

S5 Table Correlation of age-associated CpGs with chronological and predicted age for different cancer types. (PDF)

S6 Table Multivariate Cox regression model for ESCA (*p* value = 0.135). (PDF)
