# Supplementary material for: Epigenetic Aging Signatures Are Coherently Modified in Cancer
Source: PLoS Genet. 2015 Jun 25;11(6):e1005334. doi: 10.1371/journal.pgen.1005334 (PMC4482318; doi:10.1371/journal.pgen.1005334)
Supplement: S1 Fig — (PDF) [file pgen.1005334.s001.pdf]

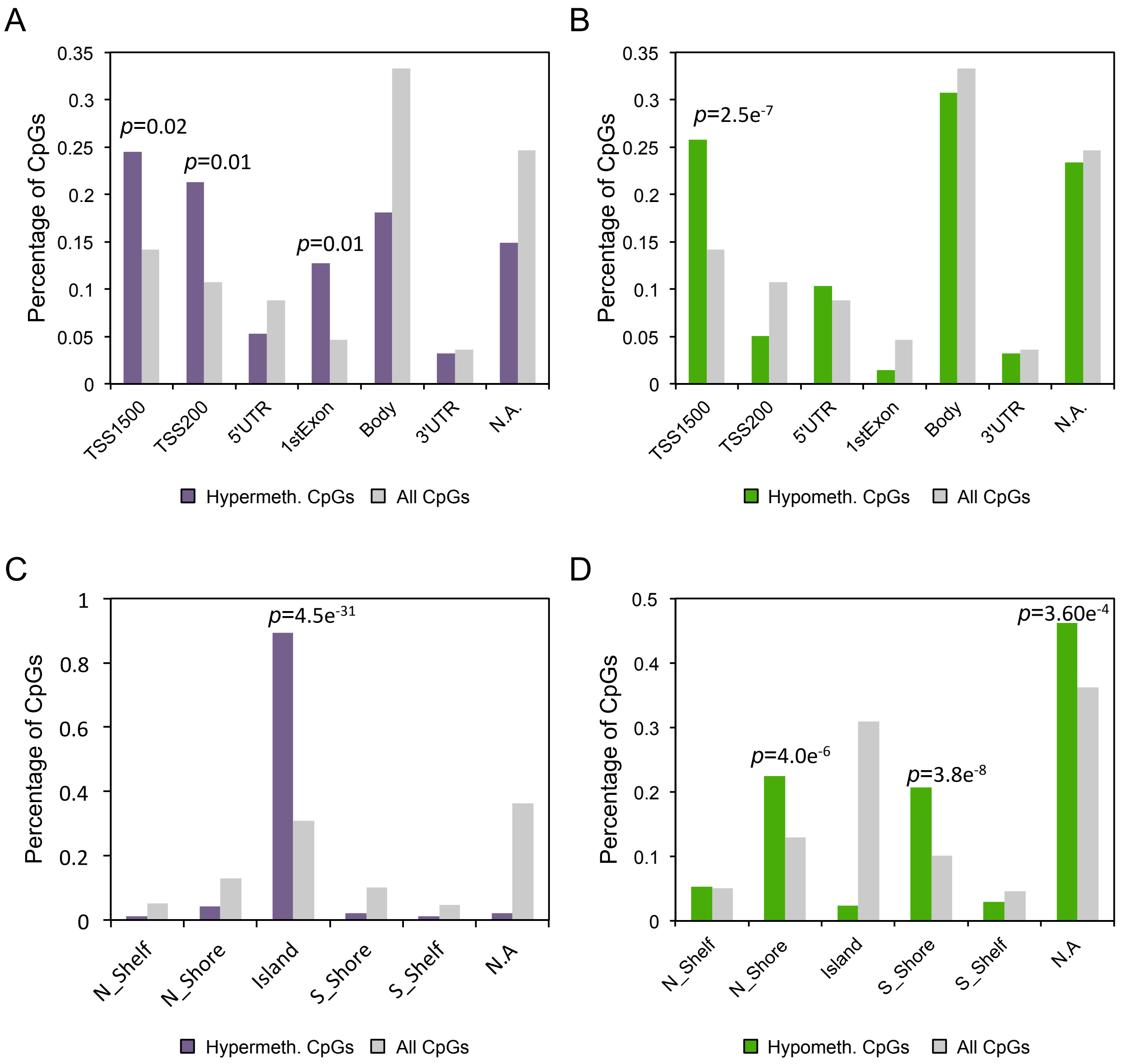

S1 Fig. Enrichment of age-associated CpGs in genomic context.

CpGs with age-associated DNAm changes were identified in the dataset of normal blood (Hannum et al., 2013). 94 hypermethylated CpGs (purple) and 338 hypomethylated CpGs (green; as also depicted in Fig 1A) were subsequently analyzed with regard to their genomic annotation. **(A-B)** Age-related hypermethylation was enriched in promoter regions (TSS1500, TSS200, and 1st Exon), whereas the hypomethylated CpGs were rather enriched in more remote promoter regions within 1500 base pairs of the transcription start site (TSS1500). **(C-D)** Age-associated hypermethylation was highly significantly enriched in CpG islands, whereas hypomethylated CpGs were enriched outside the CpG islands.
