# Supplementary material for: Epigenetic Aging Signatures Are Coherently Modified in Cancer
Source: PLoS Genet. 2015 Jun 25;11(6):e1005334. doi: 10.1371/journal.pgen.1005334 (PMC4482318; doi:10.1371/journal.pgen.1005334)
Supplement: S2 Fig — (PDF) [file pgen.1005334.s002.pdf]

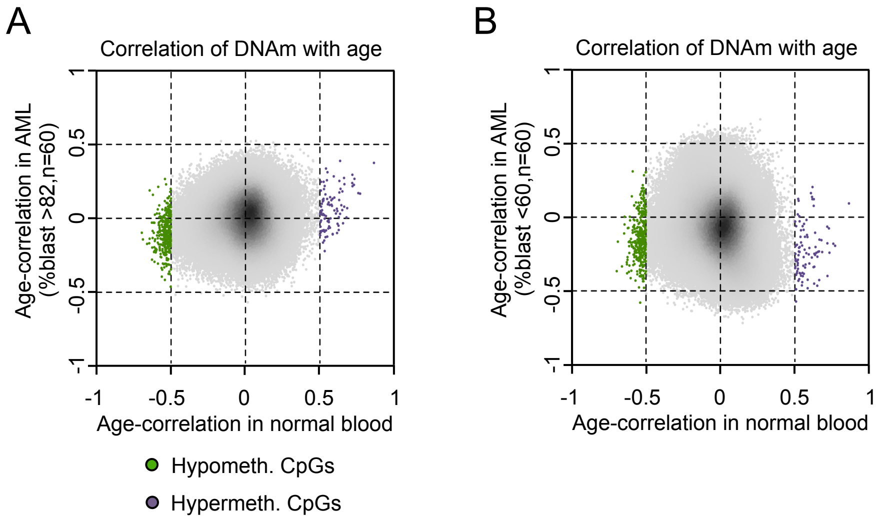

S2 Fig. Age-associated DNAm changes in AML samples with high or low blast counts.
Acute myeloid leukemia samples with either high blast counts (> 82%; **A**) or lower blast counts (< 62%; **B**) were analyzed with regard to age-associated DNAm changes (60 samples per group). Particularly in the subset with lower blast counts some CpGs revealed correlation with chronological age of the patient, but these CpGs did not overlap with age-associated changes in normal blood (as defined in Fig 1A; purple = 94 hypermethylated; green = 338 hypomethylated).
