# Supplementary material for: Epigenetic Aging Signatures Are Coherently Modified in Cancer
Source: PLoS Genet. 2015 Jun 25;11(6):e1005334. doi: 10.1371/journal.pgen.1005334 (PMC4482318; doi:10.1371/journal.pgen.1005334)
Supplement: S3 Fig — (PDF) [file pgen.1005334.s003.pdf]

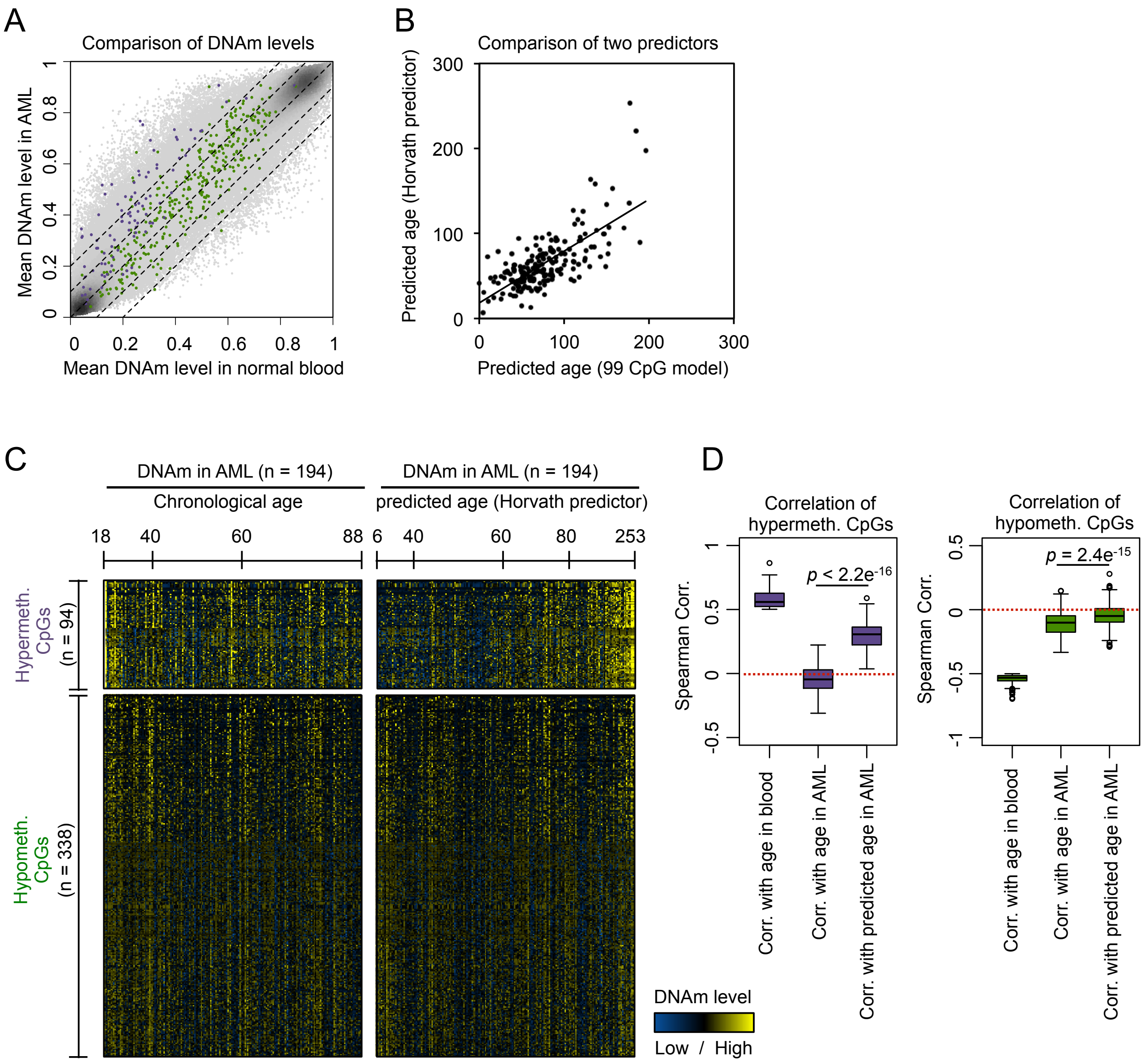

S3 Fig. Age-associated DNAm changes in AML.

(A) β-values were compared in DNAm profiles of normal blood and AML. CpGs with age-associated hypo- and hypermethylation (green and purple, respectively) are indicated as defined in Fig 1A ($\rho > 0.5$ or $\rho < -0.5$). CpGs with age-associated hypermethylation reveal higher DNAm levels in AML. **(B)** Correlation of epigenetic age predictions using either the 99 CpG model or Horvath-predictor on AML profiles. **(C)** Heatmap analysis of DNAm changes in age-associated CpGs in AML in relation to either chronological age (equivalent to Fig 1D) or epigenetic age-predictions using Horvath-predictor. **(D)** Correlation coefficients of these β-values revealed that CpGs with age-associated hypermethylation were significantly more associated with predicted age (Horvath-predictor) than with chronological age. In contrast, hypomethylated CpGs revealed only moderate correlation with chronological age and did not correlate with epigenetic age-predictions (Wilcoxon rank-sum test). Please note that results are very similar using either the 99 CpG model or Horvath-predictor, albeit the two epigenetic aging signatures are independent.
