# Supplementary material for: Epigenetic Aging Signatures Are Coherently Modified in Cancer
Source: PLoS Genet. 2015 Jun 25;11(6):e1005334. doi: 10.1371/journal.pgen.1005334 (PMC4482318; doi:10.1371/journal.pgen.1005334)
Supplement: S4 Fig — (PDF) [file pgen.1005334.s004.pdf]

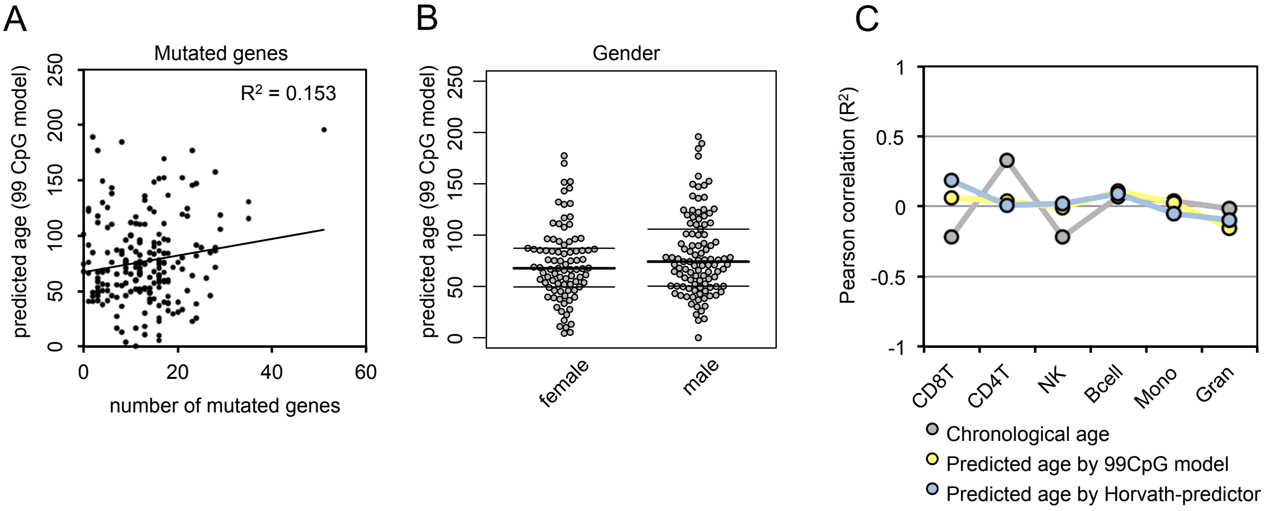

S4 Fig. Association of epigenetic age-predictions with clinical parameters in AML.

(A) The numbers of mutated genes in AML, as identified in deep sequencing data of TCGA portal, did not correlate with epigenetic age-predictions (99 CpG model). **(B)** In tendency female AML samples were predicted to be younger than male samples, which is in line with gender-specific deviations in normal blood. (**C**) The cellular composition in AML samples was estimated by bioinformatics methods described by Houseman et al. (2012; 2014). The predicted percentage of individual cellular subsets in AML samples was not clearly associated with chronological age or epigenetic age-predictions (even though the results suggest moderate increase of CD4+ T-cells in elderly patients).
