# Supplementary material for: Epigenetic Aging Signatures Are Coherently Modified in Cancer
Source: PLoS Genet. 2015 Jun 25;11(6):e1005334. doi: 10.1371/journal.pgen.1005334 (PMC4482318; doi:10.1371/journal.pgen.1005334)
Supplement: S5 Fig — (PDF) [file pgen.1005334.s005.pdf]

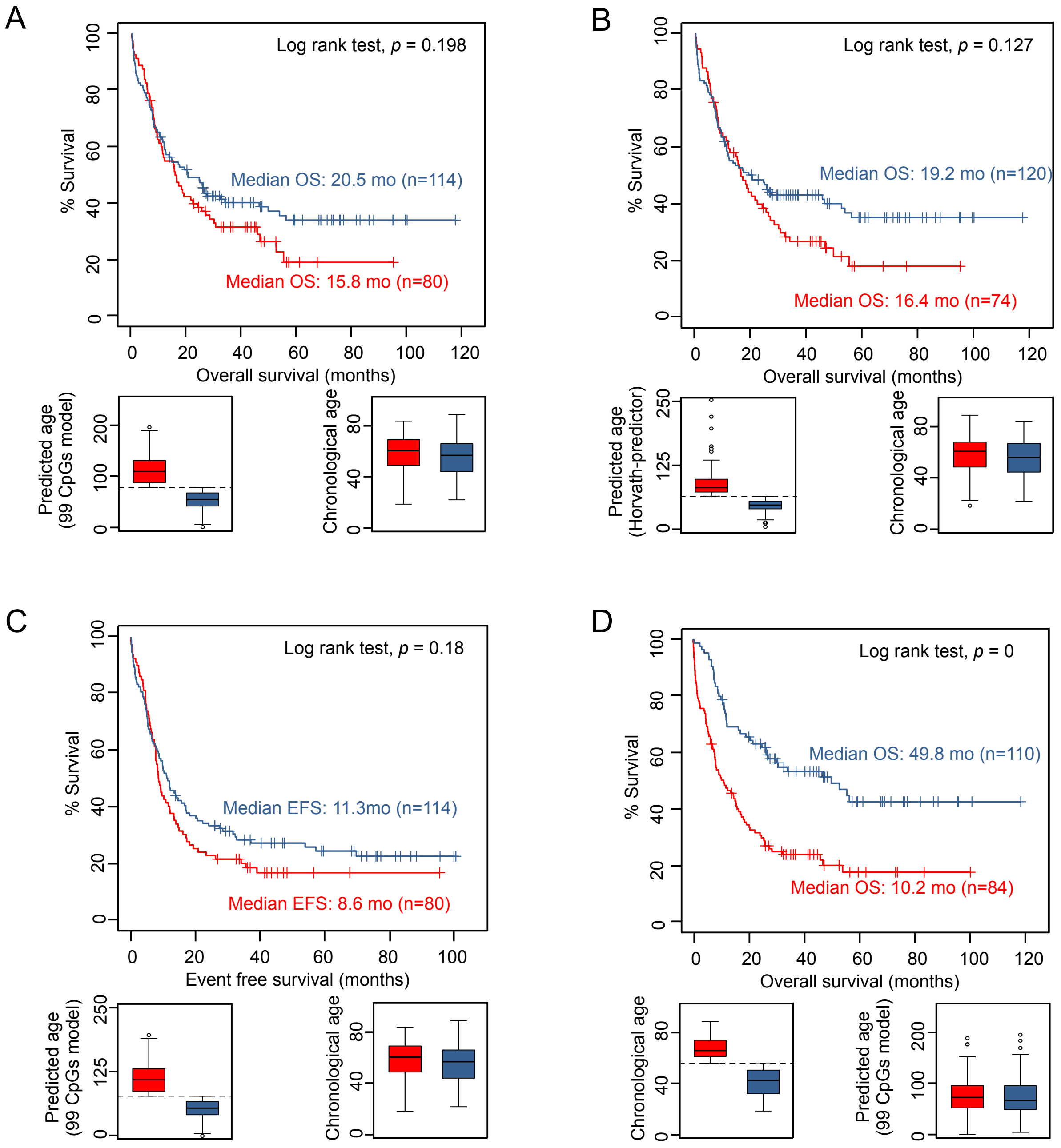

S5 Fig. Association of epigenetic age-predictions with clinical outcome in AML.

(A-B) Overall survival of AML patients was classified by mean age-predictions (**A,** 99 CpG model; **B,** Horvath-predictor; red: older than mean age-predictions of all patients; blue: younger). The Kaplan-Meier plot suggested that younger age-predictions might associate with better prognosis but this was not significant. The boxplots depict distribution of predicted age or chronological age in these two subsets. **(C)** Event free survival of AML patients classified by mean age-predictions (99 CpG model). **(D)** Overall survival of AML patients classified by mean chronological age. The Kaplan-Meier plot demonstrates significantly better prognosis of patients with younger chronological age.
