# Supplementary material for: Epigenetic Aging Signatures Are Coherently Modified in Cancer
Source: PLoS Genet. 2015 Jun 25;11(6):e1005334. doi: 10.1371/journal.pgen.1005334 (PMC4482318; doi:10.1371/journal.pgen.1005334)
Supplement: S6 Fig — (PDF) [file pgen.1005334.s006.pdf]

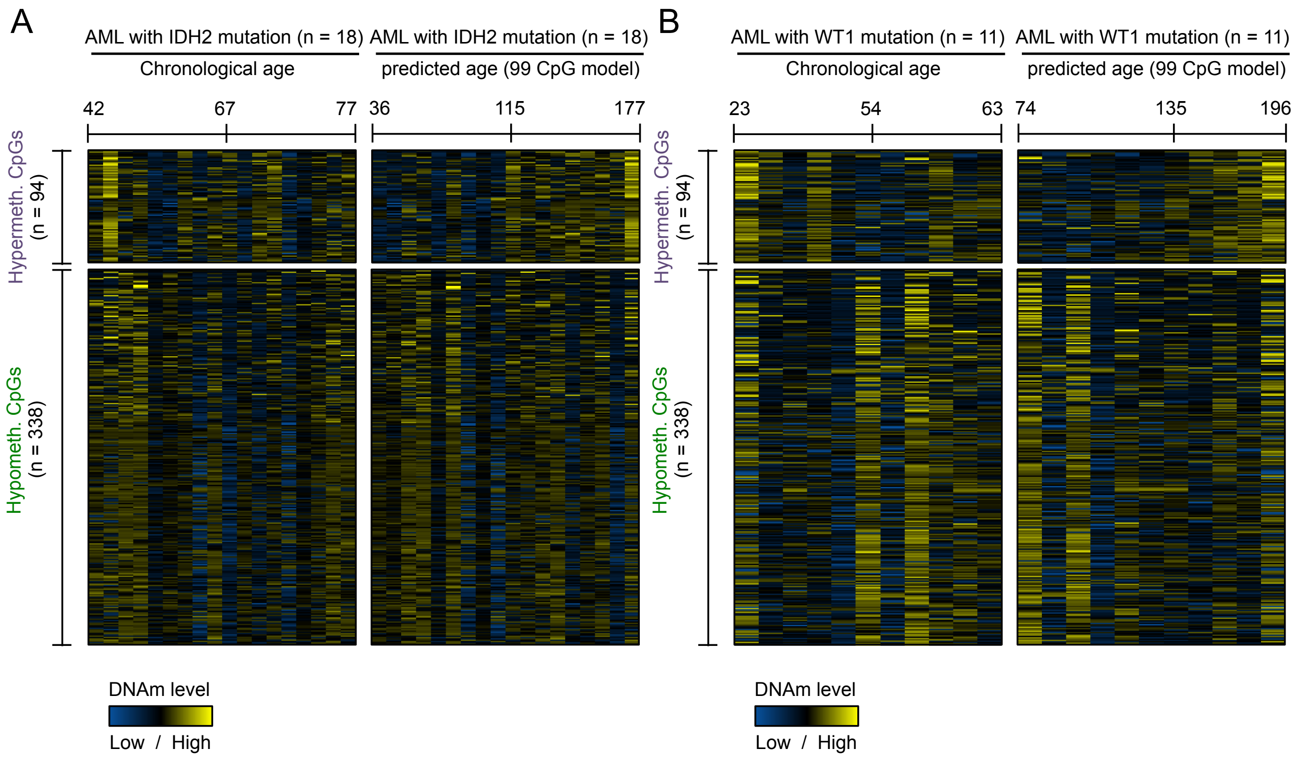

S6 Fig. DNAm of age-associated CpGs in AML samples with *IDH2* or *WT1* mutations.

These heatmaps demonstrate the age-associated CpGs that are either hyper- or hypo-methylated in normal blood (corresponding to Fig 1D). DNAm levels are exemplarily depicted for subsets of AML patients with either *IDH2* mutations **(A)** or *WT1* mutations **(B)**. Please note that CpGs with age-associated hypermethylation (purple) are also higher methylated in patients that are predicted to be older, whereas this was not observed for hypomethylated CpGs. Thus, our findings cannot simply be attributed to global DNAm levels or mutation specific signatures that are caused by different mutations.
