# Supplementary material for: Epigenetic Aging Signatures Are Coherently Modified in Cancer
Source: PLoS Genet. 2015 Jun 25;11(6):e1005334. doi: 10.1371/journal.pgen.1005334 (PMC4482318; doi:10.1371/journal.pgen.1005334)
Supplement: S7 Fig — (PDF) [file pgen.1005334.s007.pdf]

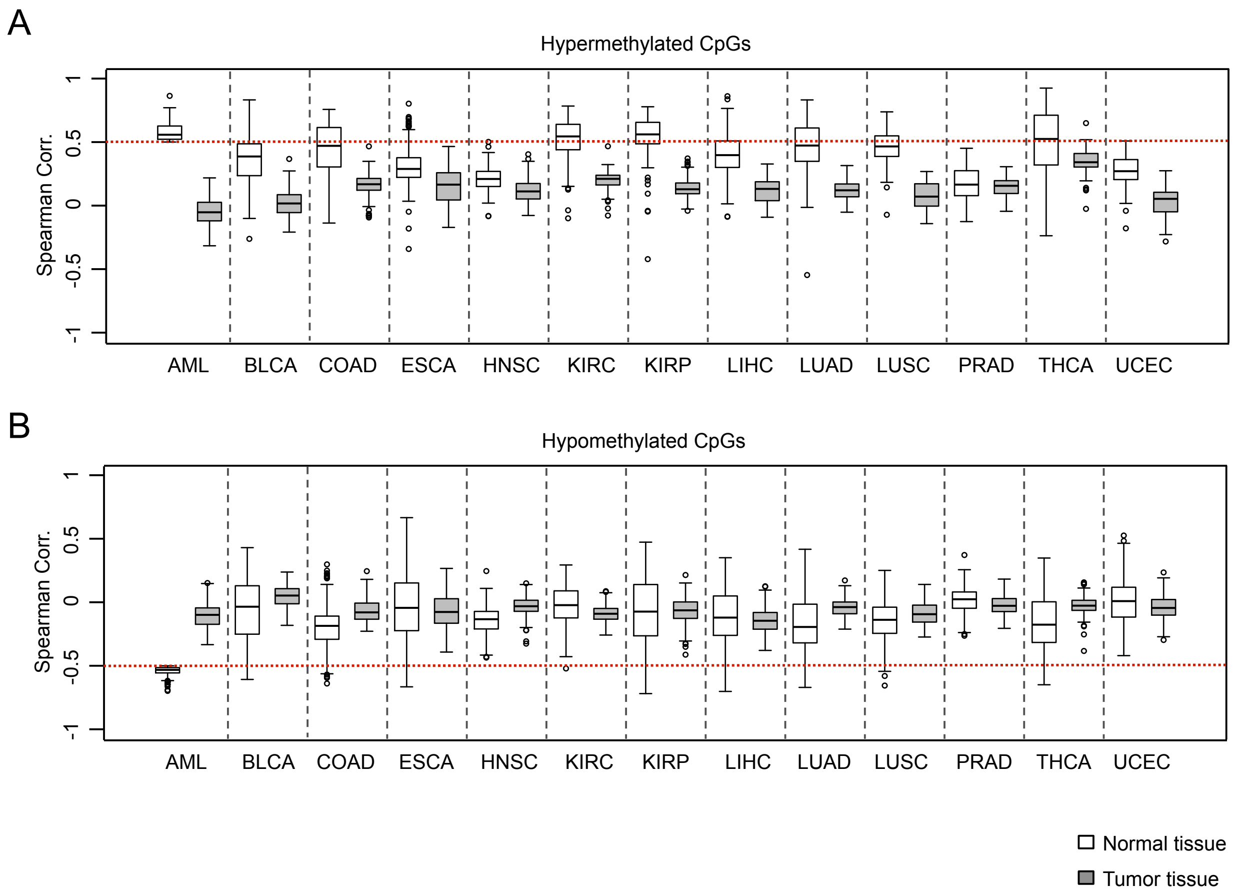

S7 Fig. Age-associated hypermethylation in blood is also reflected in other tissues.

The correlation of DNAm levels at CpGs that become either hypermethylated **(A)** or hypomethylated **(B)** upon aging in normal blood were analyzed across different tumor types and their corresponding normal controls from TCGA (short cuts for cancer types correspond to Table 1; red lines indicate thresholds for age-related CpGs in normal blood: $\rho > 0.5$ or < -0.5, respectively). Overall, hypermethylated CpGs reveal age-associated hypermethylation also in other control tissue, whereas this was hardly observed in hypomethylated CpGs. Furthermore, almost none of the CpGs correlated with chonological age in any of the cancer datasets.
