# Supplementary material for: Epigenetic Aging Signatures Are Coherently Modified in Cancer
Source: PLoS Genet. 2015 Jun 25;11(6):e1005334. doi: 10.1371/journal.pgen.1005334 (PMC4482318; doi:10.1371/journal.pgen.1005334)
Supplement: S8 Fig — (PDF) [file pgen.1005334.s008.pdf]

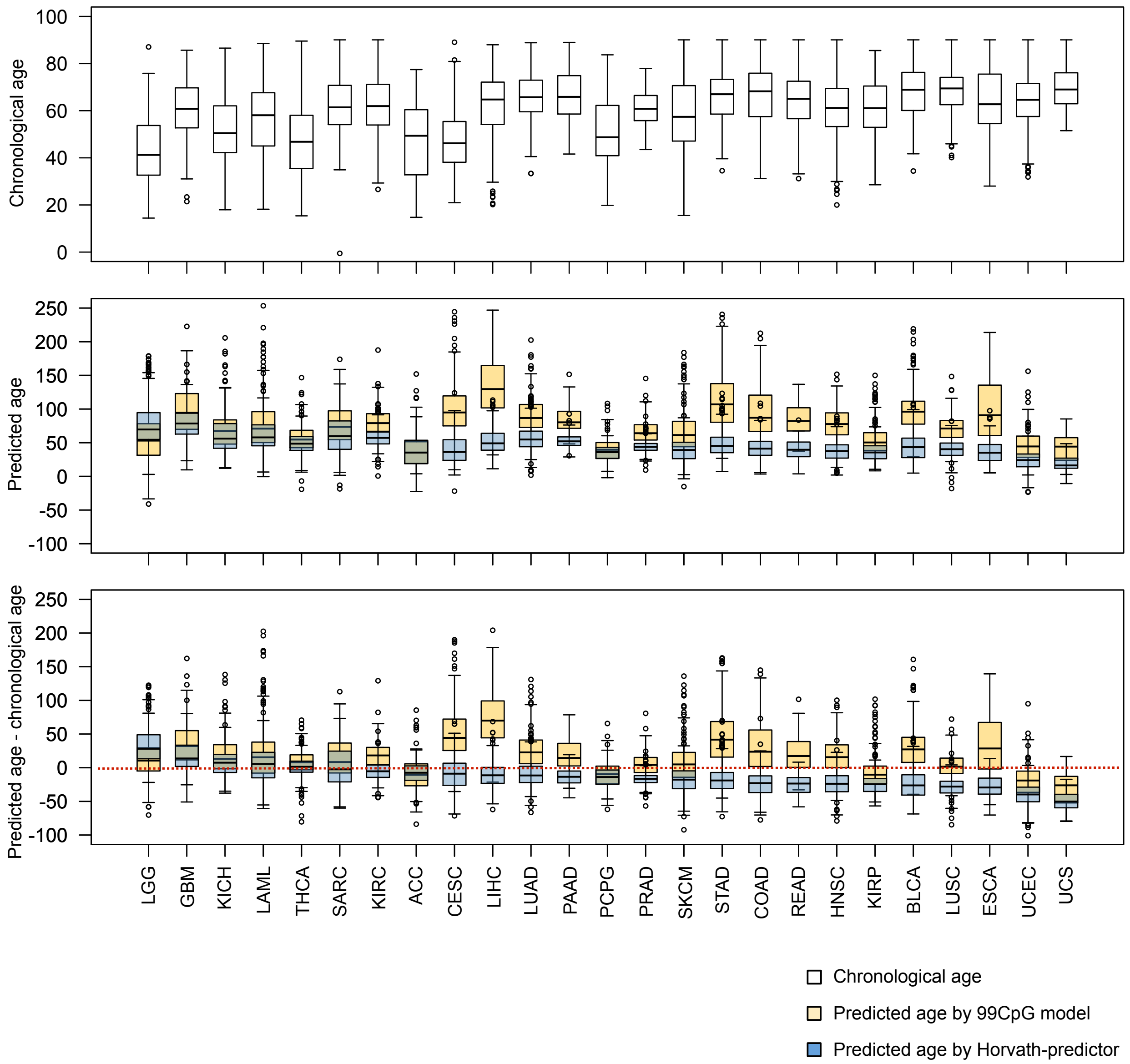

S8 Fig. Deviation of epigenetic age-predictions in various types of cancer.

Box-plots demonstrate the distribution of chronological age, predicted age (99-CpG model in yellow; Horvath-predictor in blue), and the deviation of predicted and chronological age across the different cancer types of TCGA.
