# Supplementary material for: Epigenetic Aging Signatures Are Coherently Modified in Cancer
Source: PLoS Genet. 2015 Jun 25;11(6):e1005334. doi: 10.1371/journal.pgen.1005334 (PMC4482318; doi:10.1371/journal.pgen.1005334)
Supplement: S9 Fig — (PDF) [file pgen.1005334.s009.pdf]

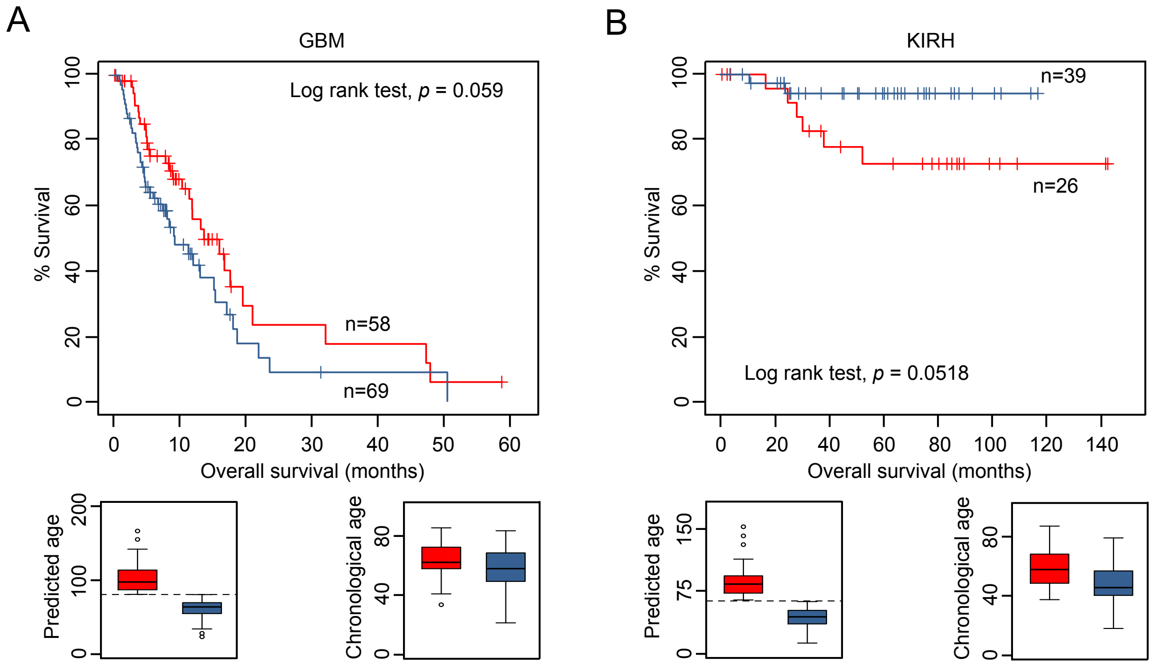

S9 Fig. Kaplan-Meier analysis of glioblastoma multiforme and kidney cancer.

Kaplan-Meier analysis of patients with **(A)** glioblastoma multiforme (GBM) and **(B)** kidney chromophobe (KIRH) stratified by mean age-predictions (Horvath-predictor; red: older than mean age-predictions of all patients; blue: younger).
