# Supplementary material for: Epigenetic Aging Signatures Are Coherently Modified in Cancer
Source: PLoS Genet. 2015 Jun 25;11(6):e1005334. doi: 10.1371/journal.pgen.1005334 (PMC4482318; doi:10.1371/journal.pgen.1005334)
Supplement: S10 Fig — (PDF) [file pgen.1005334.s010.pdf]

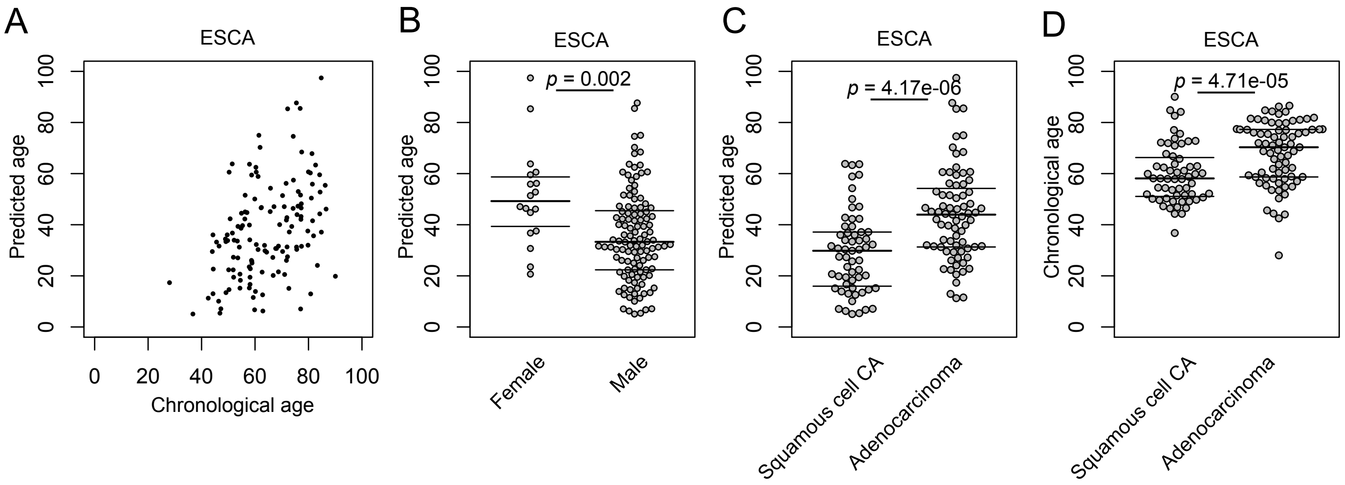

S10 Fig. Epigenetic age-predictions in esophageal carcinoma.

Epigenetic age-predictions in patients with esophageal carcinoma (ESCA) were correlated with chronological age or other clinical parameters. **(A)** Correlation of chronological age and predicted age was only moderate. **(B)** Overall, male patients were predicted to be younger than female patients. **(C)** ESCA patients with squamous cell carcinoma were predicted to be younger than those with adenocarcinoma, whereas **(D)** the differences were less pronounced with regard to chronological age.
