# Supplementary material for: Epigenetic Aging Signatures Are Coherently Modified in Cancer
Source: PLoS Genet. 2015 Jun 25;11(6):e1005334. doi: 10.1371/journal.pgen.1005334 (PMC4482318; doi:10.1371/journal.pgen.1005334)
Supplement: S1 Table — (PDF) [file pgen.1005334.s011.pdf]

S1 Table. Clinical information about AML patients (TCGA).

Parameter	Value
**Age - year**	55.0±16.0
**Male sex - no. (%)**	105 (54.1)
**Bone marrow blasts - %**	69.6±19.0
**Cytogenetic risk group - no. (%)**	
Favorable	35 (18)
Intermediate	112 (58)
Non-favorable	43 (22)
Missing data	4 (2)
**AML FAB subtype — no. (%)**	
AML with minimal maturation: M0	19 (10)
AML without maturation: M1	44 (23)
AML with maturation: M2	42 (21)
Acute promyelocytic leukemia: M3	18 (9)
Acute myelomonocytic leukemia: M4	41 (21)
Acute monoblastic or monocytic leukemia: M5	22 (11)
Acute erythroid leukemia: M6	3 (2)
Acute megakaryoblastic leukemia: M7	3 (2)
Other subtype	2 (1)
