# Supplementary material for: Epigenetic Aging Signatures Are Coherently Modified in Cancer
Source: PLoS Genet. 2015 Jun 25;11(6):e1005334. doi: 10.1371/journal.pgen.1005334 (PMC4482318; doi:10.1371/journal.pgen.1005334)
Supplement: S2 Table — (PDF) [file pgen.1005334.s012.pdf]

S2 Table. Differentially expressed genes in AML samples predicted young vs. old.

Gene Symbol	Gene Name	Gene ID	log2 fold change (below mean - above mean)	adjusted p-value
FAM127B	family with sequence similarity 127, member B	26071	-1.50	0.003
C7orf13	chromosome 7 open reading frame 13	129790	-1.28	0.011
FAM127A	family with sequence similarity 127, member A	8933	-1.93	0.011
NHLRC1	NHL repeat containing E3 ubiquitin protein ligase 1	378884	-1.02	0.013
LOC644538	small integral membrane protein 10	644538	-1.36	0.013
TEKT2	tektin 2 (testicular)	27285	-1.20	0.026
CLU	clusterin	1191	-1.56	0.035
JAG1	jagged 1	182	-1.25	0.044
DSC2	desmocollin 2	1824	-2.22	0.044
TTC12	tetratricopeptide repeat domain 12	54970	-1.13	0.046
NEXN	nexilin (F actin binding protein)	91624	1.04	0.049
