# Supplementary material for: Epigenetic Aging Signatures Are Coherently Modified in Cancer
Source: PLoS Genet. 2015 Jun 25;11(6):e1005334. doi: 10.1371/journal.pgen.1005334 (PMC4482318; doi:10.1371/journal.pgen.1005334)
Supplement: S3 Table — (PDF) [file pgen.1005334.s013.pdf]

S3 Table. Epigenetic age-predictions of AML samples with specific somatic mutations.

UID	Chronological age	Predicted age (99 CpG model)	Predicted age (Horvath predictor)	IDH2	Type
2966	57	177.35	253.35	p.R140Q	missense
2948	67	176.92	136.32	p.R140Q	missense
2805	77	152.49	87.48	p.R140Q	missense
2826	64	143.04	72.74	p.R140Q	missense
2807	68	130.80	163.22	p.R140Q	missense
2866	67	122.25	126.34	p.R140Q	missense
2891	42	121.45	112.24	p.R140Q	missense
2959	71	118.62	95.95	p.R140Q	missense
3002	68	115.63	116.48	p.R140L	missense
2934	65	110.18	127.10	p.R140Q	missense
2907	69	101.14	90.58	p.R140Q	missense
2898	69	95.96	51.73	p.R172K	missense
2877	60	81.77	70.72	p.R140Q	missense
2936	60	75.42	69.87	p.R140Q	missense
2874	59	74.17	63.67	p.R140Q	missense
2810	76	63.23	44.57	p.R140Q	missense
2821	64	42.61	25.91	p.R172K	missense
2973	67	36.39	28.51	p.R140Q	missense

UID	Chronological age	Predicted age (99 CpG model)	Predicted age (Horvath predictor)	RUNX1	Type
3009	23	195.94	197.70	p.G164fs	frame_shift_ins
2933	58	189.27	89.65	p.R162G	missense
2805	77	152.49	87.48	p.R201*,p.A142fs	nonsense,frame_shift_ins
2983	45	148.61	60.93	p.A149P	missense
2912	63	147.14	99.24	p.R162K	missense
2807	68	130.80	163.22	p.SG167in_frame_del	in_frame_del
2959	71	118.62	95.95	p.R201Q	missense
2970	34	110.47	58.72	p.P113L	missense
2907	69	101.14	90.58	e5-2	splice_site
2899	76	96.86	77.00	p.R162G	missense
2854	51	90.42	71.61	outofframe_fusion	outofframe_fusion
2978	61	84.31	92.15	p.R201*	nonsense
2949	58	84.07	79.74	p.S314fs	frame_shift_ins
2890	69	77.00	49.89	p.R201*	nonsense
2936	60	75.42	69.87	p.R320*	nonsense
2927	88	71.52	74.17	p.R201*,p.R162S	nonsense,missense
2821	64	42.61	25.91	p.D123fs	frame_shift_ins

UID	Chronological age	Predicted age (99 CpG model)	Predicted age (Horvath predictor)	TET2	Type
2845	37	107.68	45.80	p.K110fs	frame_shift_del
2978	61	84.31	92.15	e3+2,p.R318*	splice_site_ins,nonsense
3006	61	80.72	45.08	p.R254*	nonsense
2879	68	68.37	32.17	p.R18*,p.N275fs	nonsense,frame_shift_del
2923	78	64.13	44.65	p.R550*,p.Q958fs	nonsense,frame_shift_ins
2971	76	51.89	35.13	p.A1071fs,p.A665D	frame_shift_del,missense
2873	51	49.49	45.80	p.H880fs	frame_shift_del
2928	43	46.25	93.73	p.P1115fs,p.E154V	frame_shift_del,missense
2830	64	39.67	59.52	p.A183fs,p.K241fs	frame_shift_ins,frame_shift_del
2822	65	38.28	76.56	p.Q317fs	frame_shift_del
2996	75	30.73	64.97	p.R156fs,p.H670Y	frame_shift_del,missense
2964	57	29.59	47.37	p.Q916*,p.P691L	nonsense,missense
2908	81	25.92	49.07	p.R18*	nonsense
2937	35	10.90	20.02	p.F125fs	frame_shift_del
2876	45	5.18	30.64	p.Q481*,p.H672fs	nonsense,frame_shift_del
2965	60	0.00	41.71	p.P118fs	frame_shift_del

UID	Chronological age	Predicted age (99 CpG model)	Predicted age (Horvath predictor)	TP53	Type
2861	76	124.95	99.15	p.M39fs	frame_shift_ins
2938	76	105.80	81.36	p.R342fs,p.H179R	frame_shift_del,missense
2868	77	91.96	56.47	e4-1	splice_site
2904	65	86.46	77.61	p.R337C	missense
2885	71	70.96	35.02	p.H193Y	missense
2820	76	70.29	67.33	p.P223fs	frame_shift_ins
2857	54	64.50	52.73	e0+1	splice_site
2838	67	61.80	68.46	p.T125	silent
2952	60	57.64	66.94	p.E286G	missense
2829	50	57.31	59.29	p.R280G,e6-1	missense,splice_site
2878	47	56.87	36.54	p.S215G,p.V173fs	missense,frame_shift_del
2935	66	52.49	51.31	p.R248Q	missense
2941	73	49.62	53.52	p.I195S	missense
2943	70	33.31	54.01	p.R273C	missense
2813	78	32.77	55.37	p.C176Y	missense
2908	81	25.92	49.07	p.Q317*,p.C141W	nonsense,missense

UID	Chronological age	Predicted age (99 CpG model)	Predicted age (Horvath predictor)	WT1	Type
3009	23	195.94	197.70	p.A382fs	frame_shift_ins
2839	51	152.08	107.56	p.A381fs	frame_shift_ins
2913	61	136.84	158.58	e7+1	splice_site
2989	29	135.89	84.41	p.Y402fs	frame_shift_del
2844	63	111.48	44.32	p.S381fs	frame_shift_ins
2970	34	110.47	58.72	e9-1	splice_site
2930	62	96.10	58.71	p.V370fs,p.R370fs	frame_shift_ins,frame_shift_ins
2976	53	89.62	91.20	p.A382fs	frame_shift_ins
2919	54	84.94	55.21	p.H465Y	missense
2846	57	84.36	57.86	p.A382fs	frame_shift_ins
2874	59	74.17	63.67	p.R462W	missense

UID	Chronological age	Predicted age (99 CpG model)	Predicted age (Horvath predictor)	PML-RARA	Type
3007	35	75.54	44.08	PML-RARA	gene fusion
3001	31	74.77	71.66	PML-RARA	gene fusion
3012	53	70.37	62.22	PML-RARA	gene fusion
2991	40	67.67	56.67	PML-RARA	gene fusion
2998	68	65.69	61.15	PML-RARA	gene fusion
2999	62	64.48	46.63	PML-RARA	gene fusion
2982	29	55.79	52.50	PML-RARA	gene fusion
2872	42	47.99	60.65	PML-RARA	gene fusion
2863	63	45.42	32.91	PML-RARA	gene fusion
2980	50	44.69	44.05	PML-RARA	gene fusion
2840	74	41.14	55.93	PML-RARA	gene fusion
2823	61	40.87	62.53	PML-RARA	gene fusion
2803	61	39.54	42.83	PML-RARA	gene fusion
2841	51	16.92	24.40	PML-RARA	gene fusion
2897	50	13.15	47.51	PML-RARA	gene fusion
