# Supplementary material for: Epigenetic Aging Signatures Are Coherently Modified in Cancer
Source: PLoS Genet. 2015 Jun 25;11(6):e1005334. doi: 10.1371/journal.pgen.1005334 (PMC4482318; doi:10.1371/journal.pgen.1005334)
Supplement: S4 Table — (PDF) [file pgen.1005334.s014.pdf]

S4 Table. Correlation of age-predictions using 99 CpG model and Horvath-predictor for different cancer types.

Tumor type	short cut	R^2
Adrenocortical carcinoma	ACC	0.56
Bladder Urothelial Carcinoma	BLCA	0.49
Endocervical adenocarcinoma	CESC	0.59
Colon adenocarcinoma	COAD	0.58
Esophageal carcinoma	ESCA	0.61
Glioblastoma multiforme	GBM	0.71
Head and Neck squamous cell CA	HNSC	0.51
Kidney chromophobe	KICH	0.74
Renal clear cell carcinoma	KIRC	0.71
Kidney renal papillary cell CA	KIRP	0.57
Acute myeloide leukemia	LAML	0.71
Lower grade glioma	LGG	0.65
Liver hepatocellular carcinoma	LIHC	0.50
Lung adenocarcinoma	LUAD	0.74
Lung squamous cell carcinoma	LUSC	0.56
Pancreatic adenocarcinoma	PAAD	0.67
Pheochromocyt. & paraganglioma	PCPG	0.49
Prostate adenocarcinoma	PRAD	0.40
Rectum adenocarcinoma	READ	0.30
Sarcoma	SARC	0.58
Skin cutaneous melanoma	SKCM	0.64
Stomach adenocarcinoma	STAD	0.61
Thyroid carcinoma	THCA	0.56
Uterine corpus endometrial CA	UCEC	0.51
Uterine carcinosarcoma	UCS	0.55
