# Supplementary material for: Epigenetic Aging Signatures Are Coherently Modified in Cancer
Source: PLoS Genet. 2015 Jun 25;11(6):e1005334. doi: 10.1371/journal.pgen.1005334 (PMC4482318; doi:10.1371/journal.pgen.1005334)
Supplement: S5 Table — (PDF) [file pgen.1005334.s015.pdf]

S5 Table. Correlation of age-associated CpGs with chronological and predicted age for different cancer types.

Tumor Type	Short cut	CpGs with age-associated hypermethylation in control tissue: Number of CpGs (ρ > 0.05)	median corr. with chronological age in tumor tissue	median corr. with predicted age in tumor tissue	*p* value (wilcox test)	CpGs with age-associated hypomethylation in control tissue: Number of CpGs (ρ < -0.05)	median corr. with chronological age in tumor tissue	median corr. with predicted age in tumor tissue	*p* value (wilcox test)
Renal clear cell carcinoma	KIRC	1097	0.18	0.32	3.4E-174	3	-0.11	-0.06	0.70
Kidney renal papillary cell CA	KIRP	5464	0.06	0.27	0	2134	0.02	0.03	0.47
Thyroid carcinoma	THCA	1692	0.25	0.26	0.69	481	-0.03	-0.03	0.99
Lung adenocarcinoma	LUAD	2603	0.04	0.22	6.0E-294	6380	0.01	0.11	0
Liver hepatocellular carcinoma	LIHC	1362	0.05	0.20	3.7E-121	150	-0.12	0.03	7.4E-28
Bladder Urothelial Carcinoma	BLCA	6540	0.01	0.17	0	3194	-0.04	0.07	2.2E-239
Lung squamous cell carcinoma	LUSC	938	0.00	0.13	8.1E-25	266	-0.07	-0.07	0.69
Esophageal carcinoma	ESCA	16579	0.02	0.11	0	6512	0.01	0.07	3.7E-108
Pancreatic adenocarcinoma	PRAD	31	0.08	0.10	0.72	12	-0.03	-0.03	0.48
Head and Neck squamous CA	HNSC	15	0.06	0.05	1	148	0.00	-0.02	3.7E-07
Colon adenocarcinoma	COAD	16318	0.18	0.05	0	3755	-0.04	0.12	0
Uterine corpus endometrial CA	UCEC	982	0.02	0.02	0.03	158	-0.04	0.00	1.4E-06
