# Supplementary material for: Epigenetic Aging Signatures Are Coherently Modified in Cancer
Source: PLoS Genet. 2015 Jun 25;11(6):e1005334. doi: 10.1371/journal.pgen.1005334 (PMC4482318; doi:10.1371/journal.pgen.1005334)
Supplement: S6 Table — (PDF) [file pgen.1005334.s016.pdf]

S6 Table. Multivariate Cox regression model for ESCA (*p* value = 0.135).

Parameters	coef	exp(coef)	se(coef)	*p* value
Chronological age	0.0179	1.018	0.0151	0.24
Predicted Age	-0.0303	0.97	0.0132	0.021
Gender	0.1448	1.156	0.4557	0.75
Smoking	0.5288	1.697	0.4025	0.19
Histologic diagnosis	0.2628	1.301	0.375	0.48
Tumor grade	-0.025	0.975	0.1959	0.9
